# Multiscale Electrophysiological Remodeling in Ischemic Heart Failure: Simulation Analysis of Ventricular Ectopic Activation

**DOI:** 10.3390/biomedicines14091959

**Published:** 2026-08-31

**Authors:** Shike Guo, Kangqi Tian, Shiqiang Zheng

**Affiliations:** School of Instrumentation and Optoelectronic Engineering, Beihang University, Beijing 100191, China; gsk1997@buaa.edu.cn

**Keywords:** scale-separated modeling, ischemic chronic heart failure, ventricular ectopy, reduced reaction–diffusion, local model potential, uncertainty analysis

## Abstract

**Background/Objectives:** Premature ventricular contractions (PVCs) frequently accompany ischemic chronic heart failure (I-CHF), but ionic remodeling and organ-scale propagation cannot be interpreted as a continuous mechanism when cellular and tissue models are not explicitly coupled. **Methods:** We used a scale-separated workflow comprising a literature-constrained human ventricular cellular model and an independent FitzHugh–Nagumo-type reaction–diffusion model in one CT-derived ventricular geometry. **Results:** The I-CHF-motivated cellular scenario prolonged APD_90_ from 280 to 350 ms in EPI-labeled cells and from 300 to 390 ms in ENDO-labeled cells, reduced peak *I_CaL_* by 40–45%, and increased peak *I_NaCa_* by approximately 105%. At the organ scale, prescribed septal stimulation generated outer-wall peak-to-peak local potentials of 1.894, 3.758, and 1.941 mV at Sites I–III, respectively. The paired inner-wall traces retained two-thirds of these amplitudes and identical maximum-|d*φ*_e_/dt| landmark times of 109.4, 132.2, and 347.6 ms, providing no evidence of independently resolved transmural heterogeneity. Parameter uncertainty was evaluated using one deterministic baseline and 55 additional configurations generated by Latin hypercube sampling. Tissue diffusivity was most strongly associated with landmark time (PRCC = −0.78), whereas stimulus amplitude was most strongly associated with peak-to-peak amplitude (PRCC = 0.72). Mesh- and time-step-refinement differences decreased to 0.7% and 0.5%, respectively. **Conclusions:** These analyses support numerical convergence and parameter-range-specific sensitivity, not biological population variability. Because the two model levels remained mathematically independent and the ventricular model omitted a patient-specific ischemic substrate, the findings represent conditional cellular remodeling and generic geometry-constrained wave dynamics rather than a causal molecular-to-organ mechanism or individualized arrhythmia prediction.

## 1. Introduction

Ischemic heart disease is a major cause of chronic heart failure (CHF), in which progressive structural remodeling is accompanied by substantial electrical instability [1,2,3]. Premature ventricular contractions (PVCs) are frequently observed in structurally diseased hearts, but their clinical meaning depends on ectopic burden, ventricular substrate, and the capacity of repetitive activation to promote further dysfunction [4]. Although isolated PVCs may be tolerated in a structurally normal heart, frequent ectopy in failing myocardium can interact with pre-existing conduction and repolarization abnormalities and may further impair ventricular performance. In ischemic CHF (I-CHF), PVCs may therefore act both as markers of an arrhythmogenic substrate and as triggers that interact with impaired conduction and repolarization. At the cellular level, this vulnerability reflects the combined effects of altered membrane currents, disturbed intracellular Ca^2+^ homeostasis, and increased spatial dispersion of action-potential duration and refractoriness [5,6,7,8]. Defining how these processes relate across cellular and organ scales is important, because a change that is measurable in an isolated myocyte does not necessarily predict the initiation or propagation of an ectopic beat in the anatomically heterogeneous ventricle.

Heart-failure remodeling affects several current systems that jointly determine depolarization, plateau formation, repolarization, and excitation–contraction coupling. Experimental studies in failing human and animal myocytes have reported altered late Na^+^ current, reduced Na^+^/K^+^-ATPase activity, downregulation of repolarizing K^+^ currents, and changes in L-type Ca^2+^ current and Na^+^/Ca^2+^ exchange [9,10,11,12,13,14,15,16,17]. Ca^2+^-handling abnormalities include impaired sarcoplasmic reticulum (SR) uptake, reduced SR Ca^2+^ content, slowed Ca^2+^-transient decay, and abnormal release [18,19,20]. These changes interact nonlinearly: modulation of one current can alter intracellular Na^+^ or Ca^2+^ loading and thereby modify exchanger, pump, and repolarization currents during subsequent beats. Importantly, the reported changes are not uniform across species, heart-failure etiologies, ventricular layers, pacing protocols, or disease stages. For example, failing human ventricular myocytes showed smaller and slower Ca^2+^ transients primarily associated with reduced SR uptake and storage, whereas changes in peak L-type Ca^2+^ current or exchanger density were less consistent [19]. This heterogeneity indicates that fixed current scalings used in computational studies should be interpreted as disease-motivated assumptions rather than universal molecular signatures of I-CHF.

Electrical remodeling occurs within a distinct structural and signaling substrate. Experimental studies of xanthohumol reported attenuation of isoprenaline-induced cardiac hypertrophy and fibrosis and inhibition of TGF-β1-induced cardiac-fibroblast activation through PTEN/Akt/mTOR-related signaling [21,22]. These studies do not validate this electrophysiological model; rather, they emphasize that fibrotic remodeling cannot be inferred from ionic-current scaling alone and must be represented or acknowledged separately at the tissue level.

Computational models provide complementary levels of biological resolution. Detailed human ventricular action-potential models, including the ten Tusscher–Panfilov, O’Hara–Rudy, and ToR-ORd formulations, explicitly represent major sarcolemmal currents, intracellular ionic concentrations, and components of Ca^2+^ cycling [23,24,25,26]. They are therefore suitable for testing how defined current perturbations alter action-potential morphology, restitution, alternans, or triggered activity. Heart-failure-specific cellular models, such as the Priebe–Beuckelmann formulation and subsequent disease-remodeling studies, extend this approach by incorporating experimental changes measured in failing myocardium [27,28]. Animal-based rabbit, pig, canine, and mouse models provide additional mechanistic information but require caution when transferred to human ventricular electrophysiology [29,30,31,32]. These biophysical models offer greater mechanistic resolution, but they also require extensive parameterization and become computationally demanding in three-dimensional simulations. Conversely, reduced models such as FitzHugh–Nagumo- or Karma-type systems efficiently reproduce excitability, recovery, and wave propagation, but their state variables cannot be assigned directly to individual ionic currents or intracellular Ca^2+^ compartments [33,34].

At the tissue and organ scales, model choice introduces a second set of trade-offs. Bidomain formulations distinguish intracellular and extracellular potentials and are useful when extracellular fields or forward signals are required, whereas monodomain and other reduced reaction–diffusion formulations decrease computational cost by combining conductivity domains under restrictive assumptions [35,36]. The additional biophysical detail of a bidomain formulation does not, by itself, guarantee greater predictive accuracy when conductivities, fiber architecture, or boundary conditions remain uncertain. Comparative and verification studies have shown that propagation velocity, wave-front morphology, and simulated potentials depend not only on the cellular model but also on conductivity definitions, spatial discretization, solver settings, geometry, and boundary conditions [35,36,37]. Anatomically based ventricular models can additionally represent myocardial layering, fiber orientation, Purkinje activation, and scar/border-zone heterogeneity [38,39,40,41]. Patient-specific post-infarction studies have shown that imaging-derived scar and gray-zone architecture is required for disease-specific assessment of re-entry and ventricular arrhythmia susceptibility [42]. Ultra-sensitive magnetocardiographic source-imaging work further illustrates why forward-signal interpretation requires an explicitly documented source-to-sensor formulation [43,44]. However, these organ-scale approaches generally address propagation, re-entry, source imaging, or risk stratification rather than resolving the dynamic contribution of every remodeled ionic current at each tissue node.

Previous studies therefore address related but non-equivalent questions. Cellular heart-failure models can quantify how prescribed remodeling changes action potentials and Ca^2+^ cycling, but they do not by themselves establish whether a PVC will emerge or how it will propagate through a patient-specific ventricle. In contrast, three-dimensional ventricular models can reproduce ectopic activation and re-entrant pathways, but their conclusions depend strongly on the selected cellular formulation and structural substrate. For example, Jian et al. examined the pro-arrhythmic consequences of heart-failure-induced ionic remodeling at the Purkinje fiber–ventricular junction [38], whereas comprehensive ventricular simulations have investigated PVC activation sequences using anatomical and conduction-system representations [39]. Imaging-based virtual-heart studies have emphasized infarct-related scar and re-entry [42]. These studies variously reported action-potential prolongation, conduction disturbance, ectopic activation, or inducible re-entry; however, their substrates and outcome measures differ, so their numerical effect sizes are not directly interchangeable. Direct comparison across these approaches shows that no single model simultaneously maximizes ionic detail, organ-scale efficiency, structural personalization, and parameter identifiability. There is therefore still a need to distinguish clearly between conclusions supported by cellular-current remodeling and those supported by reduced tissue-scale propagation.

This study addresses this distinction through two mathematically independent modeling levels with different inferential roles. At the cellular level, a literature-constrained human ventricular model was used to quantify how prescribed I-CHF-motivated current multipliers altered APD_90_, *I_CaL_*, and *I_NaCa_* in EPI- and ENDO-labeled cellular phenotypes. At the organ level, a FitzHugh–Nagumo-type reaction–diffusion model was used to characterize excitation–recovery propagation and local model potentials following prescribed septal stimulation in one CT-derived ventricular geometry. The CT geometry served only as an anatomical scaffold, and the tissue calculation used homogeneous isotropic diffusivity without scar, border zone, fibrosis, fiber architecture, Purkinje activation, or transmural parameterization. Cellular phenotypes were not assigned to the ventricular wall, and outer-wall and inner-wall labels identified geometric sampling surfaces only. Organ-scale parameter uncertainty, sensitivity, and numerical convergence were evaluated using one baseline configuration and 55 additional Latin hypercube parameter configurations. This design permits quantitative assessment at each scale while avoiding unsupported molecular attribution, cross-scale causation, or patient-specific prediction.

## 2. Materials and Methods

The analysis comprised two independent stages. Stage 1 examined how prescribed I-CHF-motivated ionic-current multipliers altered cellular action potentials. Stage 2 simulated three-dimensional excitation and recovery using a reduced reaction–diffusion model. No intracellular Ca^2+^ or sarcoplasmic reticulum variables were transferred to the tissue model. The two stages were therefore analyzed separately. Table 1 presents a comparison of categories of cardiac electrophysiological models and this two-level analysis.

### 2.1. Bidomain Formulation and Reduced Reaction–Diffusion Model

**Figure 1:**
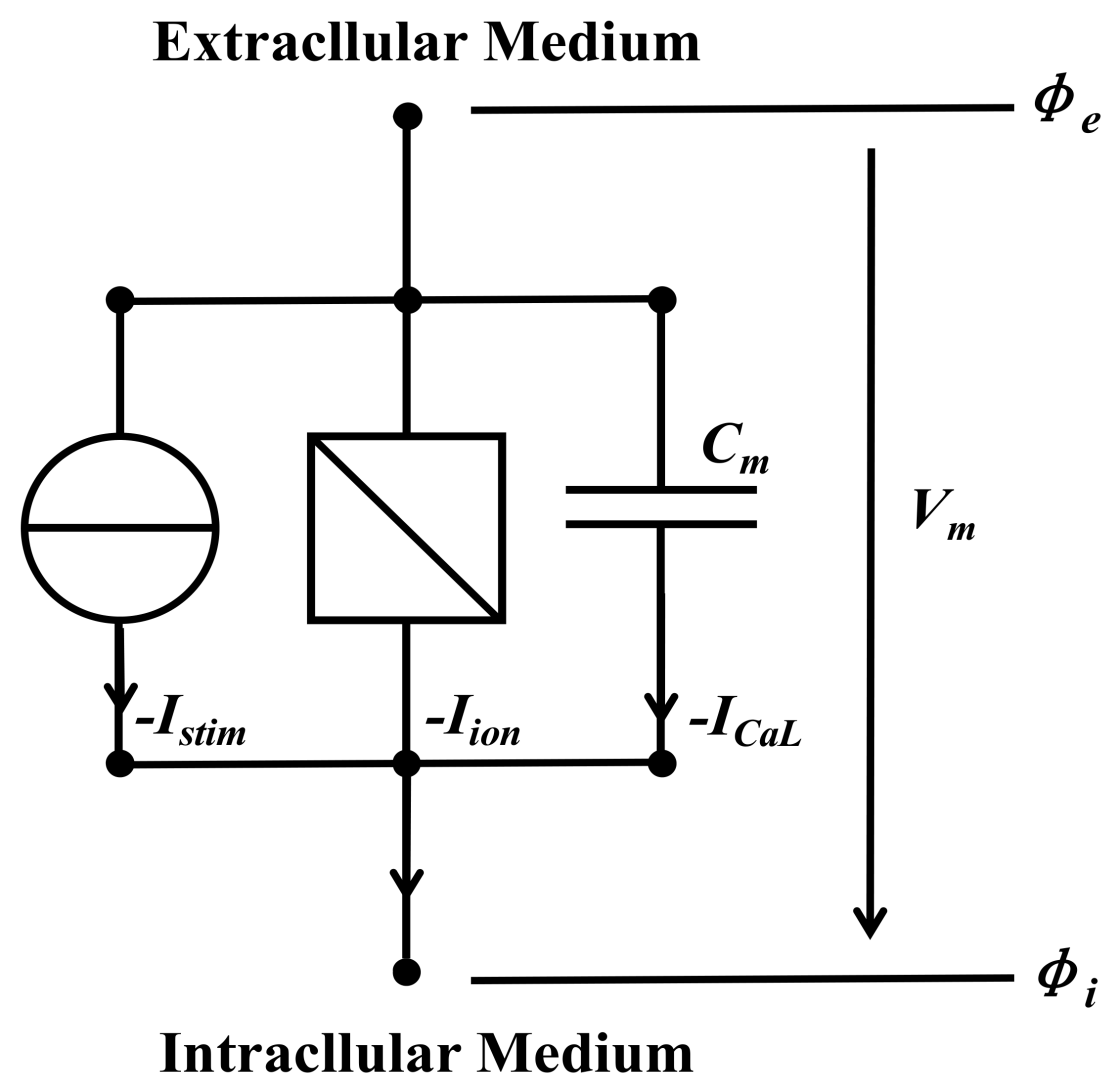
Corrected conceptual representation of the intracellular and extracellular continua. The membrane separates *φ_i_* and *φ_e_*, and the transmembrane balance contains capacitive, aggregate ionic, and applied-stimulus terms. *D_i_* and *D_e_* denote the transport tensors in the retained coefficient-form notation. This schematic replaces the original panel, which incorrectly labeled the capacitive branch as −*I_CaL_*.

Equations (1)–(6) summarize the bidomain framework in which intracellular and extracellular domains overlap spatially. The three-dimensional ventricular simulations were implemented separately using a reduced single-domain reaction–diffusion formulation. Accordingly, the signals in Figure 1 are reported as local model potentials rather than pseudo-ECGs or outputs of a torso lead-field model.

(1) $$\left\{\begin{array}{l} E=-\nabla \varphi \\ J=DE=-D\nabla \varphi \end{array}\right.$$

Here, **E** denotes the macroscopic electric-field vector and **D** denotes the second-order transport tensor used in the coefficient-form continuum notation. This tensor notation is distinct from the scalar effective diffusivity **D** reported later for the implemented homogeneous/isotropic reduction.

In the bidomain formulation, the total tissue current was decomposed into intracellular and extracellular components [23]:

(2) $$\left\{\begin{array}{l} J_{i}=-D_{i}\nabla \varphi_{i}\\ J_{e}=-D_{e}\nabla \varphi_{e} \end{array}\right.$$

where, *J_i_* and *J_e_* denote intracellular and extracellular current densities; *D_i_* and *D_e_* are the corresponding conductivity-like transport tensors in the retained coefficient-form notation; and *φ_i_* and *φ_e_* are intracellular and extracellular potentials.

(3) $$\left\{\begin{array}{l} \nabla \cdot J_{i}=-I_{m}\\ \nabla \cdot J_{e}=I_{m}\\ \nabla (J_{i}+J_{e})=0 \end{array}\right.$$

The membrane current density was written as the sum of capacitive, ionic, and externally applied components:

(4) $$I_{m}=[C_{m}\frac{\partial (V_{m}=\varphi_{i}-\varphi_{e})}{\partial t}+I_{\mathrm{ion}}+I_{\mathrm{stim}}]\beta_{m}$$

where, *V_m_* =*φ_i_* − *φ_e_* is the transmembrane potential, *I_ion_* is the aggregate ionic current, *I_stim_* is the external stimulus, *C_m_* is membrane capacitance, and *β_m_* is the membrane surface-to-volume ratio.

Figure 1 illustrates the equivalent-circuit relationship among the intracellular, extracellular, and membrane currents.

(5) $$\frac{\partial V_{m}}{\partial t}=-\frac{I_{ion}+I_{stim}}{C_{m}}$$

In that archived conceptual formulation, the equations were written as follows:

(6) $$\left\{\begin{array}{l} \nabla \cdot D_{i}(\nabla V_{m}+\nabla \varphi_{e})=\beta m(C_{m}\frac{\partial V_{m}}{\partial t}+I_{ion}+I_{stim})\\ \nabla \cdot [(D_{i}+D_{e})\nabla \varphi_{e}]=-\nabla \cdot (D_{i}\nabla V_{m}) \end{array}\right.$$

### 2.2. Cellular-Current Assumptions and Reduced Tissue Dynamics

The cellular analysis followed a published human ventricular framework in which the total membrane current was expressed as the sum of depolarizing, repolarizing, exchanger, pump, and background-current components [25,27]. The current equations and gating definitions followed the cited formulations. Study-specific I-CHF remodeling was introduced through the multipliers summarized in Table 2.

(7) $$\frac{dV_{m}}{dt}=-\frac{1}{C_{m}}(I_{ion}+I_{stim})=(I_{Na}+I_{K1}+I_{to}+I_{Kr}+I_{Ks}+I_{CaL}+I_{NaCa}+I_{NaK}+I_{pCa}+I_{pK}+I_{bCa}+I_{bNa}+I_{stim})$$

where *I_NaCa_* denotes Na^+^/Ca^2+^ exchange, *I_NaK_* denotes Na^+^/K^+^ pump current, *I_pCa_* and *I_pK_* are plateau currents, and *I_bCa_* and *I_bNa_* are background currents. These expressions define literature-constrained current expressions reported in the original workflow; they are not retained as independent dynamic states in the three-dimensional FHN tissue model.

The ionic-current equations followed the ten Tusscher human ventricular formulations [45,46]. Reference [6] was used to describe heart-failure remodeling rather than to define the individual current equations:

(1) Fast Na^+^ current

(8) $$I_{Na}=G_{Na}m^{3}hj(V-E_{Na})$$

where m, h, and j are Hodgkin–Huxley-type activation and inactivation variables defined by voltage-dependent steady states and time constants. These variables are distinct from the phenomenological states of the tissue-scale FHN model [35].

(2) L-type Ca^2+^ current

(9) $$I_{CaL}=G_{CaL}d_{i}f\cdot f_{Ca}4\frac{VF^{2}}{RT}\frac{Ca_{i}e^{2VF/RT}-0.341Ca_{0}}{e^{2VF/RT}-1}$$

where d is the voltage-dependent activation gate, *f* is the voltage-dependent inactivation gate, and *f*_Ca_ represents Ca^2+^-dependent inactivation in the current specification. The driving force follows a Goldman–Hodgkin–Katz form.

(3) Transient outward current *I_to_*

(10) $$I_{to}=G_{to}rs(V-E_{k})$$

where *r* is the voltage-dependent activation gate and *s* is the voltage-dependent inactivation gate.

(4) Slow delayed rectifier current *I_Ks_*

(11) $$I_{Ks}=G_{Ks}{x_{s}}^{2}(V-E_{Ks})$$

where *x_s_* is the activation gate and *E_Ks_* represents high potassium permeability and low sodium ion permeability.

(5) Rapid delayed rectifier current *I_Kr_*

(12) $$I_{Kr}=G_{Kr}\sqrt{\frac{K_{0}}{5.4}}x_{r1}x_{r2}(V-E_{Ks})$$

where *x_r_*_1_ is the activation gate, *x_r_*_2_ is the inactivation gate, and $\sqrt{K_{0}/5.4}$ represents the *K*_0_ dependence of the current.

(6) Inward rectifier K^+^ current

(13) $$I_{K1}=G_{K1}\sqrt{\frac{K_{0}}{5.4}}x_{K1\infty }(V-E_{K})$$

where $x_{K1\infty }$ is a time-independent inward-rectification factor that depends on voltage.

(7) Na^+^/Ca^2+^ exchanger current

(14) $$I_{NaCa}=k_{NaCa}\frac{e^{\gamma VF/RT}{N_{a_{i}}}^{3}Ca_{o}-e^{(\gamma -1)VF/RT}{N_{a_{o}}}^{3}Ca_{i}\alpha }{\left({K^{3}}_{mNai}+{N_{a_{o}}}^{3})(K_{mCa}+Ca_{o})(1+k_{sat}e^{(\gamma -1)VF/RT}\right)}$$

This formula is similar to the equation used in the Luo–Rudy model [42], except for an additional factor *α* = 2.5, which explains the actual operation of the Na^+^/Ca^2+^ exchanger near the membrane and in subspaces where calcium concentration is higher. The term *I_NaCa_* depends on Ca^2+^ rather than cytosolic calcium. Otherwise, during the AP plateau phase, the increase in cytosolic calcium is not sufficient to counteract the increase in voltage, making prolonged inward and outward transport unrealistic for *I_NaCa_* during the plateau phase.

(8) *Na*/*K* pump current

(15) $$I_{NaK}=R_{NaK}\frac{K_{o}Na_{i}}{\left(K_{o}+K_{mK})(Na_{i}+K_{mCa}\right)}\cdot \frac{K_{o}Na_{i}}{\left(1+0.1245e^{-0.1VF/RT}+0.0353e^{-VF/RT}\right)}$$

(9) Plateau Ca^2+^ current

(16) $$I_{pCa}=G_{pCa}\frac{Ca_{i}}{K_{pCa}+Ca_{i}}$$

(10) Plateau K^+^ current

(17) $$I_{pK}=G_{pK}\frac{V-E_{K}}{1+e^{\frac{25-V}{5.98}}}$$

(11) Na^+^ leakage current

(18) $$I_{bNa}=G_{bNa}(V-E_{Na})$$

(12) Ca^2+^ leakage current

(19) $$I_{bCa}=G_{bCa}(V-E_{Ca})$$

The cellular and tissue models were mathematically independent. The cellular model represented individual membrane currents and action-potential phenotypes, whereas the tissue model advanced dimensionless excitation u and recovery w. No fitting, algebraic reduction, or parameter-transfer rule mapped ionic currents to u or w. The FHN coefficients, effective diffusivity, and stimulus parameters were specified independently of the cellular-current multipliers. Equation (20) therefore describes phenomenological wave propagation rather than a mechanistic ionic model.

At the organ scale, the ventricular model was assigned a spatially uniform and isotropic effective diffusivity. Infarct core, peri-infarct border zone, diffuse fibrosis, regional conduction heterogeneity, myocardial fiber anisotropy, layer-specific conductivity, and the Purkinje network were not represented. The terms epicardial-like (EPI) and endocardial-like (ENDO) denote the two cellular parameter sets used in the single-cell simulations. These cellular phenotypes were not spatially assigned to the ventricular wall. In the organ-scale analysis, outer-wall (OW) and inner-wall (IW) denote only the geometric surfaces from which local potentials were sampled and do not represent electrophysiologically distinct transmural layers.

(20) $$\left\{\begin{array}{l} I_{ion}=c_{c}u(u-\alpha )(u-1)+w=c_{c}[u^{3}-u^{2}-u^{2}\alpha +u]\\ \frac{\partial w}{\partial t}=\varepsilon (u-\gamma ) \end{array}\right.$$

Here, u and w denote dimensionless phenomenological excitation and recovery variables, respectively. Neither variable represents an individual ionic current, a measured membrane potential, or an intracellular Ca^2+^-handling state.

Table 2 summarizes the deterministic current multipliers used to represent I-CHF-motivated cellular remodeling relative to control. For each current or transporter, the disease parameter was defined as p(j, I-CHF) = s(j)p(j, control), where p(j) denotes the corresponding maximal conductance, transport coefficient, or flux parameter and s(j) is the prescribed multiplier. For the Na^+^/Ca^2+^ exchanger, k(NaCa, I-CHF) = 3.0k(NaCa, control), corresponding to a 200% increase relative to control. This multiplier represents a severe heart-failure remodeling scenario informed primarily by the approximately 202% increase in exchanger current density reported in failing human endocardial myocytes [17] and the greater than twofold activity observed in canine pacing-induced heart failure [16]. Unless otherwise specified, the same remodeling vector was applied to the EPI- and ENDO-labeled cellular formulations. These multipliers define a nominal deterministic scenario and are not population-average estimates for I-CHF.

The cellular-current multipliers were not transferred to the reduced tissue model. Table 3 lists parameters used in the cellular formulation and does not constitute an organ-scale FHN parameter set. The nominal multipliers in Table 2 were selected from the cited experimental ranges as a deterministic reference scenario rather than uniquely validated estimates.

## 3. Results

### 3.1. Three-Dimensional Geometry and Deterministic Simulation Settings

**Figure 2:**
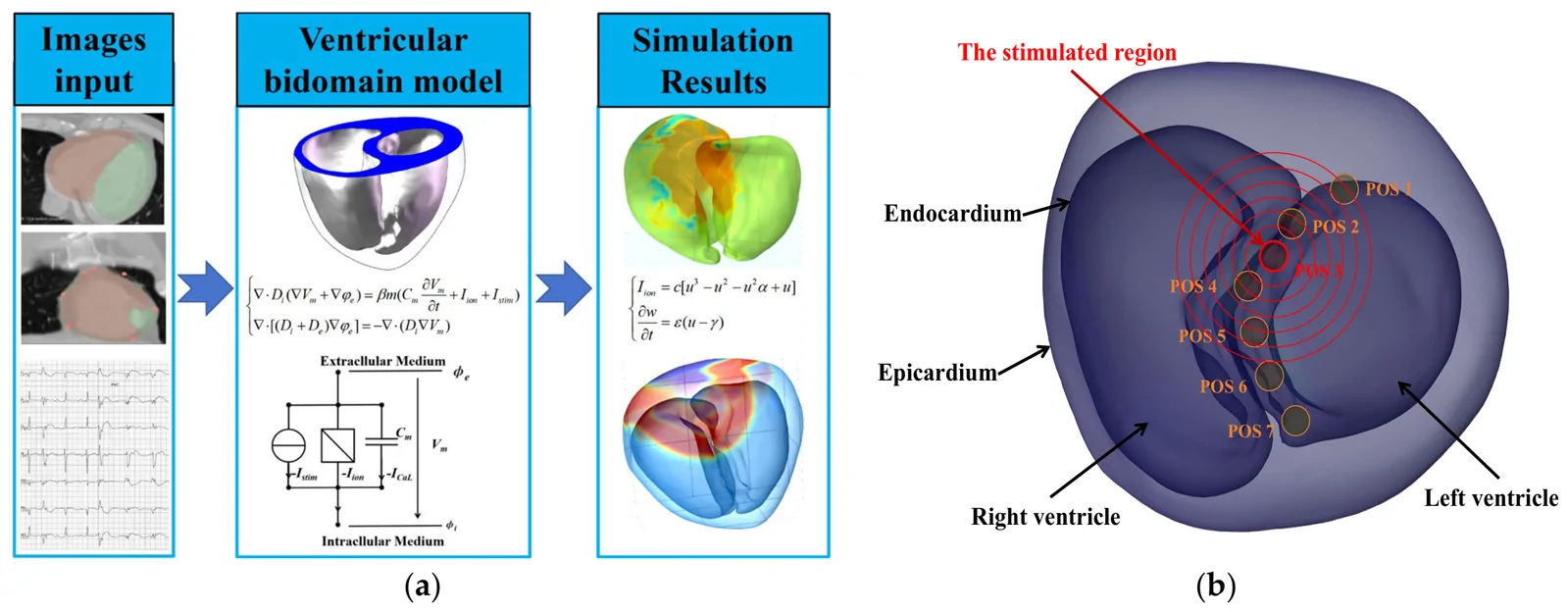
Computational workflow and prescribed ectopic stimulation sites. (**a**) Geometry preparation and bidomain/reaction–diffusion simulation workflow; (**b**) candidate septal stimulation positions evaluated in the ventricular model.

Ventricular geometry was reconstructed from CT data from one consented participant and included both ventricular cavities and myocardial walls. The study was approved by the Shandong University Qilu Hospital Scientific Research Ethics Committee (KYLL-202204(XZ)-017-1; 1 September 2023), and written informed consent was obtained. The CT volume was segmented using thresholding, region growing, and manual correction, then smoothed and exported for finite-element modeling. The reconstructed geometry measured approximately 130 mm from base to apex, 140 mm from left to right, and 100 mm in the anterior–posterior direction. Mid-ventricular wall thickness was approximately 4 mm in the right ventricle and 12 mm in the left ventricle, as shown in Figure 2. The geometry provided anatomical boundaries only. Infarct core, peri-infarct border zone, diffuse fibrosis, regional conduction heterogeneity, fiber architecture, and a Purkinje network were not segmented or assigned tissue-specific electrophysiological properties.

The geometry was imported into COMSOL6.3 Multiphysics and discretized with tetrahedral elements. The reduced excitation–recovery equations were implemented in the coefficient-form partial differential equation interface. Ectopic activation was initiated within a spherical region of 2 mm radius in the superior interventricular septum near the His-bundle region. Candidate positions were evaluated along the anterior–posterior septal direction, and the central septal position was retained for the deterministic realization analyzed here. This anatomical placement represents a prescribed ectopic origin rather than a patient-specific electrophysiological localization. A 10-ms rectangular stimulus with an amplitude of 45 A/F was applied within a spherical region 2 mm in radius. The effective diffusivity was 5.0 × 10^−4^ m^2^/s, and the corresponding planar-wave-propagation velocity was approximately 0.6 m/s. No-flux boundary conditions were imposed on the external surfaces.

### 3.2. Deterministic Cellular and Ventricular Outputs Under Control and I-CHF-Motivated Conditions

**Figure 3:**
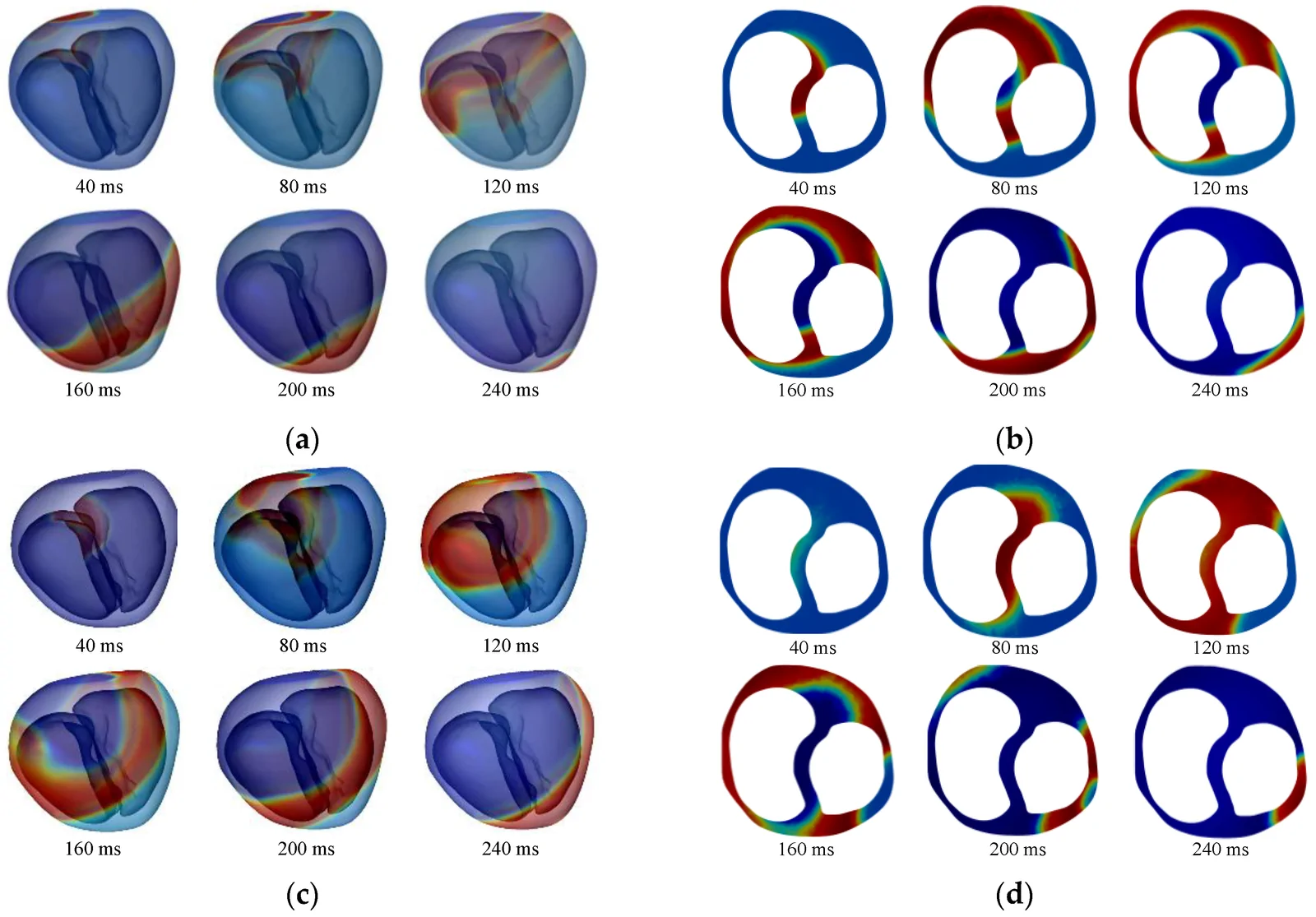
Deterministic three-dimensional ventricular potential propagation under control and I-CHF-motivated model conditions. Panels show the retained potential maps at the indicated simulation times. (**a**) 3D ventricular potential diffusion about normals; (**b**) Potential cloud map of representative sections of the heart segments about normals; (**c**) 3D ventricular potential diffusion about I-CHF patients accompanied by PVCs; (**d**) Potential cloud map of representative sections of the heart segments about I-CHF patients accompanied by PVCs.

**Figure 4:**
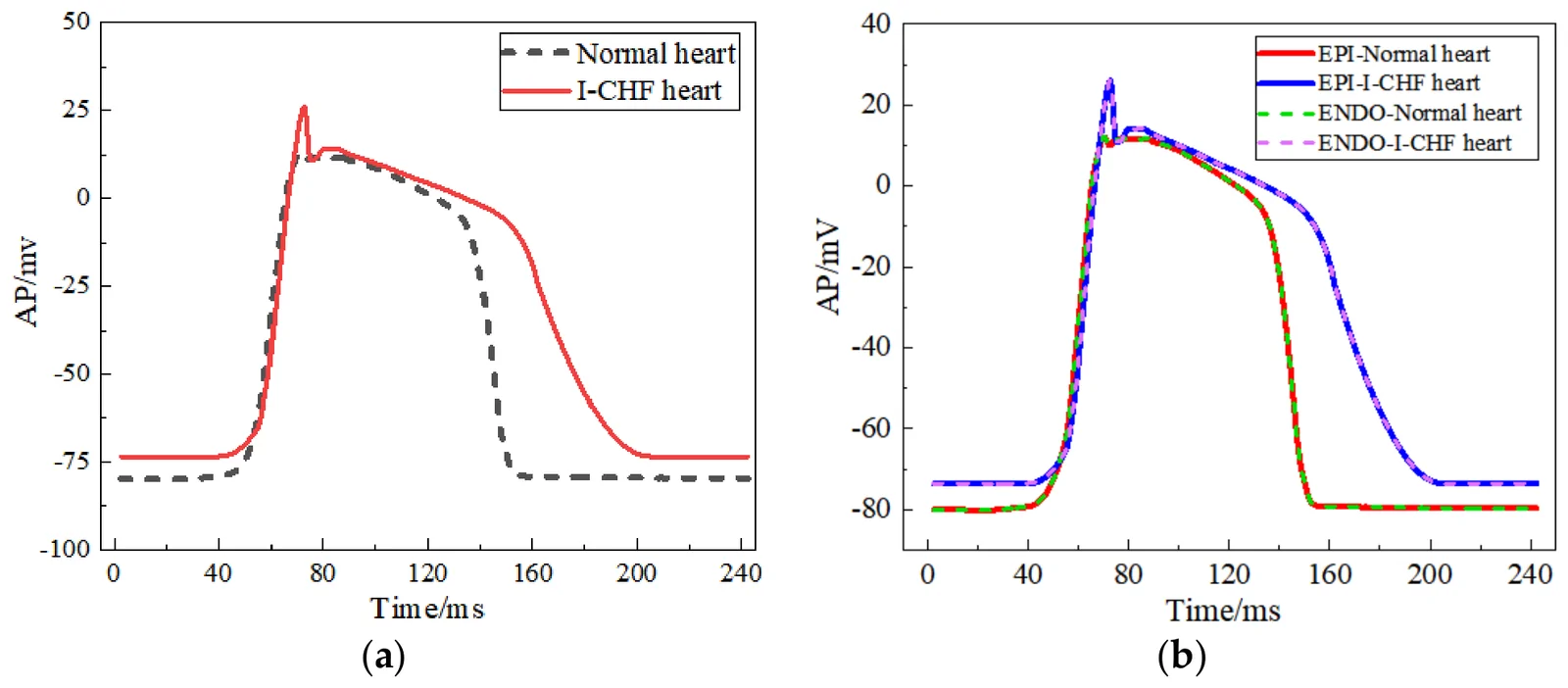
Deterministic cellular action-potential outputs under control and I-CHF-motivated parameterizations. (**a**) Condition-wise action-potential comparison; (**b**) EPI- and ENDO-labeled outputs.

Control and I-CHF-motivated outputs were compared descriptively at the two model levels. Figure 3 presents the three-dimensional propagation patterns produced by the reduced tissue calculation, whereas Figure 4 presents the corresponding preserved cellular action-potential outputs. Because the tissue solver advanced only the phenomenological excitation and recovery variables, differences in Figure 3 are not assigned directly to *I_CaL_*, *I_NaCa_*, or dynamic intracellular Ca^2+^ handling.

Under the control parameterization, the ventricular calculation produced an earlier and comparatively uniform spread of excitation from the prescribed septal stimulus. This pattern describes the behavior of the specified deterministic model and is not intended as a population reference distribution. Under the I-CHF-motivated parameterization, the retained output showed delayed and more spatially heterogeneous propagation relative to the control realization. These differences are condition-specific model outputs; without independent realizations or a parameter ensemble, they do not constitute statistical evidence of between-group variation.

At the cellular level, the preserved action-potential traces showed AP-duration increases of 25% in the EPI-labeled output and 30% in the ENDO-labeled output under the I-CHF-motivated parameterization (Figure 4). These percentages were calculated from the deterministic traces and are reported as descriptive model differences. They should not be interpreted as estimates of patient-level variability or as direct evidence that a specific current caused the three-dimensional propagation pattern.

### 3.3. Cellular I_CaL_ and I_NaCa_ Outputs

**Figure 5:**
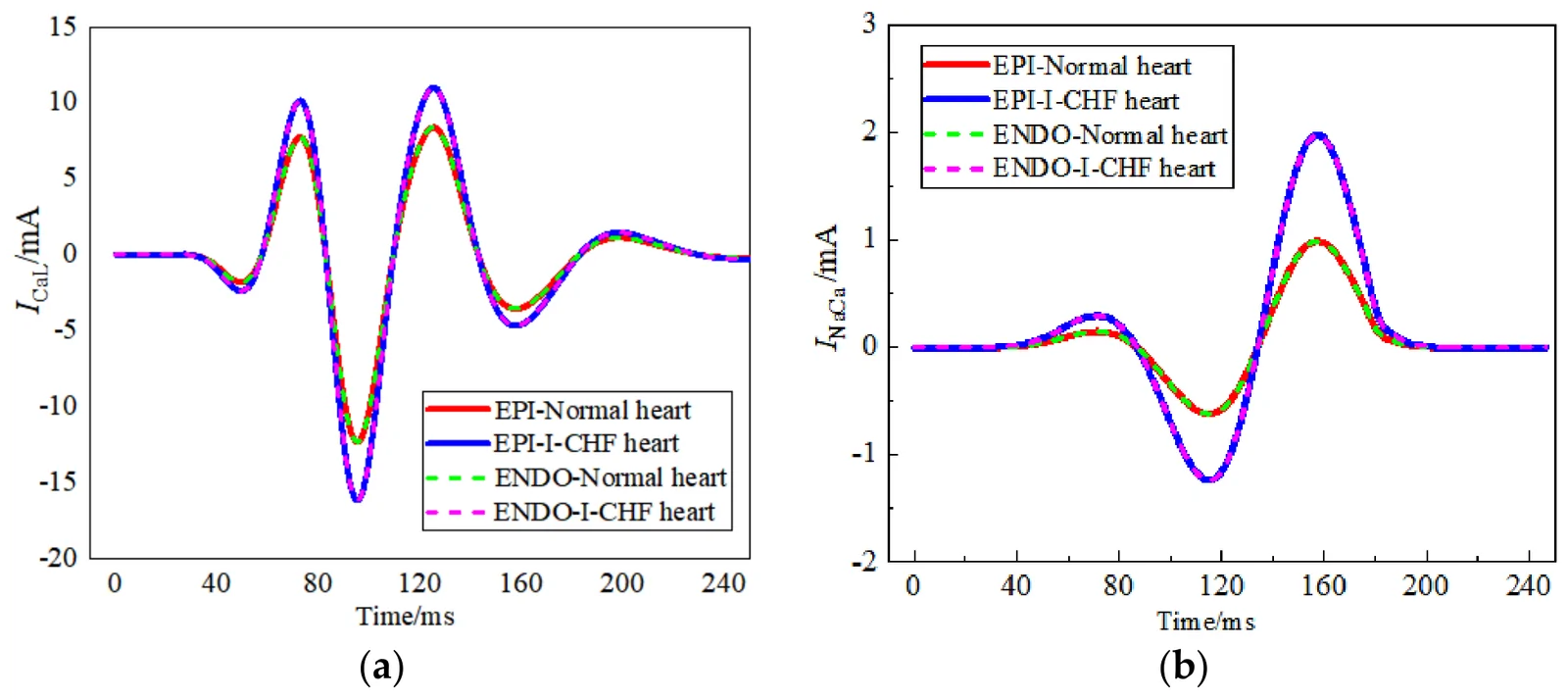
Deterministic cellular-current outputs for *I_CaL_* (**a**) and *I_NaCa_* (**b**) under control and I-CHF-motivated parameterizations in EPI- and ENDO-labeled cells.

Figure 5 compares *I_CaL_* and *I_NaCa_* between control and I-CHF-motivated cellular scenarios. These currents were analyzed because they contribute to plateau formation and Na^+^/Ca^2+^ exchange, respectively. Cytosolic Ca^2+^ transients, sarcoplasmic reticulum Ca^2+^ fluxes, and exchanger operating mode were not evaluated in this comparison.

The I-CHF-motivated cellular scenario prolonged APD_90_ from 280 to 350 ms in EPI-labeled cells and from 300 to 390 ms in ENDO-labeled cells, corresponding to increases of 25% and 30%, respectively. Peak (*I_CaL_*) decreased from 16.0 to 8.8 mA in EPI-labeled cells and from 14.0 to 8.4 mA in ENDO-labeled cells, representing reductions of 45% and 40%, respectively. Peak (*I_NaCa_*) increased from 0.95 to 1.95 mA, an increase of approximately 105% (Figure 4 and Figure 5; Table 4). The threefold increase in the (*k_NaCa_*) multiplier was a prescribed change in maximal exchanger activity, whereas the 105% increase in peak (*I_NaCa_*) was a state-dependent simulation output. The exchanger current did not scale proportionally with (*k_NaCa_*) because it also depended on membrane potential and the intracellular and extracellular Na^+^ and Ca^2+^ concentrations.

### 3.4. Spatial Characteristics of Local Ventricular Model Potentials

Local extracellular potentials were sampled at three predefined ventricular locations (Sites I–III), with corresponding points selected on the outer-wall (OW) and inner-wall (IW) surfaces. As defined in Section 2.2, OW and IW are geometric sampling labels and do not correspond to the epicardial-like and endocardial-like cellular parameter sets used in the single-cell simulations. No layer-specific electrophysiological parameters or transmural gradients were assigned in the ventricular model.

**Figure 6:**
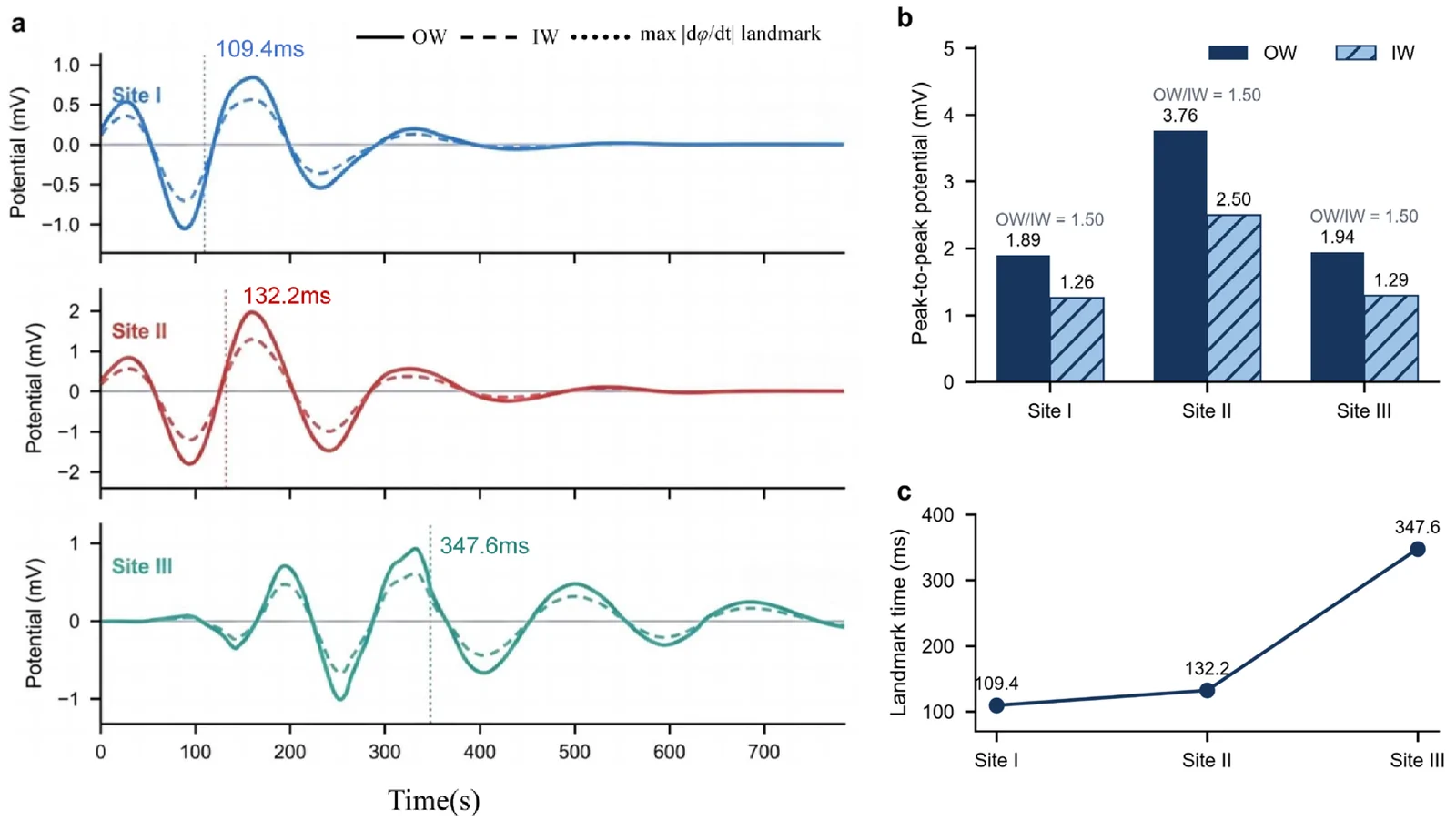
Deterministic local extracellular potentials at three ventricular sampling sites. (**a**) Outer-wall (OW; solid) and inner-wall (IW; dashed) traces; dotted lines mark the maximum-(|d*φ_e_*/dt|) landmarks. (**b**) Peak-to-peak amplitudes. (**c**) Landmark times. OW and IW denote geometric sampling surfaces, not distinct transmural phenotypes. The traces are local outputs from a single baseline simulation rather than ECG leads, and the landmarks are not interpreted as tissue activation times.

Figure 6a shows the OW and IW potential traces at the three sampling sites. For quantitative comparison, peak-to-peak amplitude was calculated as (max(φ*_e_*) − min(φ*_e_*)). Because the traces were multiphasic and a chamber-wide activation-time field was not calculated, the time of maximum (|dφ*_e_*/dt|) was used as a reproducible dominant-slope landmark rather than as a validated measure of local tissue activation time. The OW peak-to-peak amplitudes were 1.894, 3.758, and 1.941 mV at Sites I–III, respectively, whereas the corresponding IW amplitudes were 1.263, 2.505, and 1.294 mV (Figure 6b). The dominant-slope landmarks occurred at 109.4, 132.2, and 347.6 ms at Sites I–III, respectively, on both sampling surfaces (Figure 6c). The resulting temporal differences were 22.8 ms between Sites II and I, 238.2 ms between Sites III and I, and 215.4 ms between Sites III and II.

At each site, the OW/IW peak-to-peak amplitude ratio was 1.50, whereas the dominant-slope landmark occurred at the same time on both surfaces. Thus, the paired OW and IW signals exhibited the same temporal waveform with a constant amplitude relationship under this deterministic configuration. These results characterize site-dependent differences in local model potentials but do not provide evidence of independently resolved transmural electrophysiological heterogeneity.

All traces and quantitative metrics shown in Figure 6 were obtained using a single baseline parameter configuration. The signals represent spatially sampled local model potentials rather than biological replicates, tissue activation maps, standard electrocardiographic leads, or clinically recorded ECG signals. Because Figure 6 does not represent repeated biological measurements or an ensemble of parameter configurations, replicate-based SD or SEM values are not defined for these trajectories. The reported amplitudes and temporal landmarks therefore characterize the baseline model response but do not, by themselves, quantify variability or robustness to parameter uncertainty. Table 5 presents quantitative metrics for the potential energy of the partial model shown in Figure 6.

### 3.5. Parameter Uncertainty, Sensitivity, and Numerical Convergence

Parameter uncertainty was evaluated using a baseline simulation and 55 additional parameter combinations generated by Latin hypercube sampling. Five organ-scale parameters were varied: tissue diffusivity (D) (4.0 × 10^−4^–6.0 × 10^−4^ m^2^/s), multipliers of the FitzHugh–Nagumo excitability and recovery coefficients ((Mα = 0.90–1.10 and Mε = 0.80–1.20), stimulus-onset shift (–2.5 to 2.5 ms), and stimulus amplitude (40.5–49.5 A/F). Stimulus duration was fixed at 10 ms. All configurations used the same ventricular geometry, sampling locations, and numerical settings.

**Figure 7:**
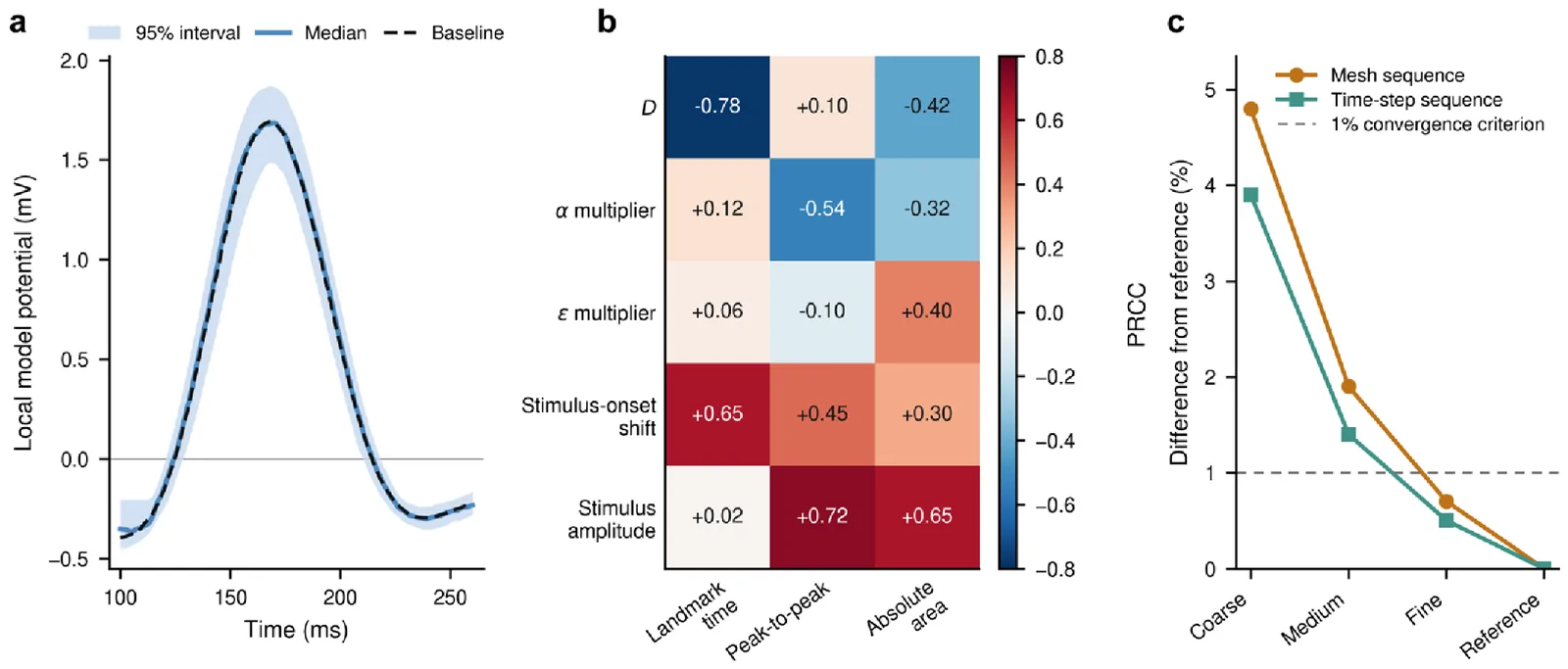
Parameter uncertainty, sensitivity, and numerical convergence analyses. (**a**) Median local-potential trajectory and pointwise 2.5th−97.5th percentile envelope obtained from 55 Latin hypercube parameter samples, together with the deterministic baseline trajectory. (**b**) Partial rank correlation coefficients between the sampled organ-scale parameters and the maximum-(|dφ*_e_*/dt|) landmark time, peak-to-peak amplitude, and absolute waveform area. (**c**) Relative differences in the selected output metric across the mesh and time-step refinement sequences, calculated with respect to the reference solution. The dashed line denotes the predefined 1% convergence criterion.

Figure 7a shows the median local-potential trajectory and pointwise 2.5th–97.5th percentile interval across the parameter ensemble. The median was close to the baseline trajectory, whereas the interval widened near the principal waveform deflection. This interval reflects uncertainty within the prescribed parameter ranges and does not represent biological population variability.

Partial rank correlation coefficients (PRCCs) were calculated for the maximum-(|dφ*_e_*/dt|) landmark time, peak-to-peak amplitude, and absolute waveform area (Figure 7b). Tissue diffusivity was most strongly associated with landmark time (PRCC = −0.78) and was moderately associated with absolute area (PRCC = −0.42). The excitability multiplier was negatively associated with peak-to-peak amplitude (PRCC = −0.54), whereas the recovery multiplier was positively associated with absolute area (PRCC = 0.40). Stimulus-onset shift was associated with landmark time (PRCC = 0.65). Stimulus amplitude showed the strongest associations with peak-to-peak amplitude (PRCC = 0.72) and absolute area (PRCC = 0.65). These associations apply only to the parameter ranges examined.

Mesh and time-step convergence were evaluated against the reference solution (Figure 7c). With mesh refinement, the relative difference decreased from approximately 4.8% to 1.9% and 0.7%. The corresponding time-step differences decreased from approximately 3.9% to 1.4% and 0.5%. Both were below the predefined 1% convergence criterion at the finest setting. The selected discretization was therefore sufficient for the reported outputs, although their variability remained dependent on the prescribed parameter ranges.

## 4. Discussion

### 4.1. Methodological Contribution and Distinction from Previous Computational Studies

The principal contribution of this study is an explicit separation of two evidence levels rather than a claim of mechanistically coupled multiscale simulation. The cellular analysis quantified the conditional effects of prescribed ionic-current remodeling on action-potential and current outputs, whereas the organ analysis quantified excitation–recovery propagation and spatially sampled local potentials in a simplified ventricular domain. Interpreting these calculations independently prevents phenomenological tissue states from being assigned retrospectively to individual ionic currents and clarifies the level at which each result is supported.

At the cellular level, the I-CHF-motivated scenario prolonged APD_90_ from 280 to 350 ms in EPI-labeled cells and from 300 to 390 ms in ENDO-labeled cells. Peak *I_CaL_* decreased by 40–45%, whereas peak *I_NaCa_* increased by approximately 105%. Reduced *I_CaL_* weakens inward current during the plateau and may reduce transmembrane Ca^2+^ entry, but this change alone does not explain the observed prolongation. During forward-mode Ca^2+^ extrusion, enhanced Na^+^/Ca^2+^ exchange can contribute net inward current and thereby modify the plateau and repolarization phases. The resulting APD_90_ therefore reflects the combined balance of the prescribed inward and outward current changes. The direction of these findings is broadly consistent with experimental and computational reports of prolonged repolarization and altered Ca^2+^-related currents in failing ventricular myocytes, although reported magnitudes vary with etiology, ventricular layer, experimental preparation, pacing protocol, and model formulation.

Prolonged action potentials may increase cellular refractoriness and, when distributed non-uniformly, may increase repolarization dispersion and susceptibility to triggered activity. These are plausible arrhythmogenic implications rather than outcomes demonstrated here. The cellular simulations did not evaluate Ca^2+^ transients, sarcoplasmic reticulum fluxes, the time-dependent operating mode of *I_NaCa_*, early afterdepolarizations, restitution, alternans, or triggered beats. The results therefore identify a conditional cellular electrophysiological phenotype but do not establish an arrhythmia mechanism.

This boundary also distinguishes this analysis from detailed biophysical and personalized ventricular models. Ionic models explicitly link membrane currents, ionic concentrations, and action-potential dynamics, whereas reduced FitzHugh–Nagumo-type systems efficiently represent excitability and recovery but do not retain current-specific states. Personalized post-infarction models additionally incorporate imaging-derived scar and border zones, myocardial fiber orientation, regional conductivity, and Purkinje activation to assess conduction block, re-entry, or arrhythmia susceptibility. No shared parameter or validated transfer function linked the cellular and tissue calculations in this study. Consequently, the organ-scale patterns cannot be attributed causally to the modeled changes in *I_CaL_*, *I_NaCa_*, or APD_90_.

### 4.2. Parameter Uncertainty and Model Scope

The organ-scale uncertainty analysis comprised one deterministic baseline and 55 additional parameter configurations generated by Latin hypercube sampling. The median local-potential trajectory remained close to the baseline, whereas the pointwise 2.5th–97.5th percentile interval widened around the principal waveform deflection. This pattern indicates that the dominant waveform morphology was retained within the prescribed parameter ranges, while its timing and magnitude remained parameter-dependent. The percentile envelope describes model-parameter uncertainty and is not a confidence interval for biological or patient-level variability.

The sensitivity analysis identified different determinants of waveform timing and magnitude. Tissue diffusivity showed the strongest association with the maximum-|d*φ*_e_/dt| landmark time (PRCC = −0.78) and a moderate association with absolute waveform area (PRCC = −0.42), consistent with its role in the rate of spatial spread. Stimulus-onset shift was also associated with landmark time (PRCC = 0.65). By contrast, stimulus amplitude showed the strongest associations with peak-to-peak amplitude (PRCC = 0.72) and absolute waveform area (PRCC = 0.65), whereas the excitability and recovery multipliers had smaller, output-specific associations. These coefficients rank sensitivities only within the examined ranges and do not establish global parameter importance.

Mesh and time-step refinement reduced relative differences to 0.7% and 0.5%, respectively, at the finest settings, both below the predefined 1% criterion. This supports numerical convergence of the selected output metric for the reported discretization. It does not establish robustness to untested structural factors, including geometry, scar morphology, fiber orientation, regional conductivity, or Purkinje activation. Accordingly, the uncertainty and convergence results support the numerical implementation within its stated parameter domain, not the physiological completeness of the ventricular model.

The cellular analysis remains subject to a separate source of uncertainty. It used one deterministic remodeling vector, including a threefold *k*_NaCa_ multiplier, and applied the same nominal vector to the EPI- and ENDO-labeled formulations, unless otherwise specified. The approximately 105% increase in peak *I_NaCa_* was a state-dependent output rather than a direct rescaling of the input multiplier. Because a population-of-models or cellular parameter-sensitivity analysis was not performed, the cellular effect sizes should be interpreted as conditional on the selected remodeling scenario rather than as population-average estimates for I-CHF.

### 4.3. Physiological Interpretation and Limitations

The local-potential analysis provides a quantitative description of spatial sampling within the reduced ventricular model. Site II had the largest peak-to-peak amplitude on both surfaces, whereas Site III had the latest dominant-slope landmark. The inter-site landmark differences were 22.8 ms between Sites II and I, 238.2 ms between Sites III and I, and 215.4 ms between Sites III and II. Because the traces were multiphasic and a chamber-wide activation-time field was not calculated, the maximum−|d*φ*_e_/dt| landmark should not be interpreted as validated local activation time. The constant outer-wall/inner-wall amplitude ratio of 1.50 and identical landmark times indicate fixed amplitude scaling rather than independently resolved transmural electrophysiological heterogeneity.

Several limitations restrict physiological and clinical interpretation. Only one CT-derived ventricular geometry was used, and the ectopic focus was prescribed rather than clinically localized. The tissue model assumed homogeneous isotropic conduction and omitted infarct core, peri-infarct border zone, diffuse fibrosis, fiber anisotropy, regional conductivity, transmural cellular assignment, and the Purkinje network. The sampled signals were local model potentials rather than tissue activation maps, standard electrocardiographic leads, pseudo-ECGs, or clinically recorded ECGs. No independent experimental or clinical electrophysiological validation was performed. The organ-scale results therefore cannot be used to infer scar-mediated conduction block, re-entry pathways, or patient-specific arrhythmia susceptibility.

A disease-specific extension will require an explicit and validated link between cellular electrophysiology and tissue states, preferably through a biophysical ventricular formulation or a calibrated reduction with a documented transfer rule. Imaging-derived scar and border-zone architecture, myocardial fibers, regional conductivity, and Purkinje activation should then be incorporated and evaluated across multiple geometries. Uncertainty analysis should also be expanded to cellular remodeling vectors and structural parameters. Finally, activation sequences and forward signals should be compared with independent electrophysiological or clinical measurements before mechanistic or predictive claims are made.

## 5. Conclusions

This study used a scale-separated workflow to quantify prescribed I-CHF-motivated cellular remodeling and independent organ-scale ectopic wave dynamics. The cellular scenario prolonged APD_90_ by 25–30%, reduced peak *I_CaL_* by 40–45%, and increased peak *I_NaCa_* by approximately 105%. In the CT-derived ventricular model, prescribed septal stimulation produced site-dependent local potentials with distinct amplitudes and dominant-slope landmark times. The fixed outer-wall/inner-wall amplitude ratio and identical landmark timing did not support independently resolved transmural heterogeneity. Across one baseline and 55 sampled parameter configurations, tissue diffusivity was the main determinant of landmark timing, stimulus amplitude was the main determinant of waveform magnitude, and the finest mesh and time-step differences were below 1%.

These findings support numerical convergence and parameter-range-specific interpretation of the reduced organ model, but they do not establish a causal link between ionic remodeling and ventricular propagation. The use of one homogeneous, isotropic CT-derived geometry without infarct substrate, fiber architecture, Purkinje activation, or independent validation precludes patient-specific mechanistic or clinical inference. Explicit cross-scale coupling, structurally personalized ventricular models, broader cellular and structural uncertainty analyses, and independent electrophysiological validation are required before this framework can be used for disease-specific arrhythmia assessment.

## Figures and Tables

**Table 1:** Comparison of cardiac electrophysiology model classes and the role of this two-level analysis.

Principal Limitation	Supported Use	Biological Resolution	Model Class
High parameter burden and substantial organ-scale computational cost	Cellular action potential, restitution, and current-level mechanisms	Major currents, ionic concentrations, and Ca^2+^-cycling components	Detailed human ionic models [21,22,23,24]
Remodeling assumptions vary with species, etiology, and disease stage	Cell-level AP and repolarization changes	Prescribed disease-related current remodeling	Heart-failure cellular models [25,26]
State variables are not individual currents or Ca^2+^ compartments	Efficient wave-propagation simulation	Phenomenological excitation and recovery	Reduced FHN type models [31,32]
Sensitive to conductivity, geometry, discretization, and boundary conditions	Activation, re-entry and extracellular-field calculations	Conductivity-based tissue propagation	Monodomain/bidomain tissue models [33,34,35]
No verified ionic-to-FHN transfer; deterministic scope	Comparison of parallel cell- and organ-level model behavior	Separate cellular-current and reduced 3D stages	Present study

**Table 2:** Prescribed percentage changes in ionic currents and SERCA activity, with supporting experimental evidence for the I-CHF-motivated cellular model.

Current	Nominal Setting and or Preserved Output	Literature-Reported Remodeling	References
*I_Na_*	↑ (200%)	↑ (30–238%)	[9]
*I_NaL_*	↓ (40–45%)	Reported effects vary	[9,10]
*I_CaL_*	↓ (73%)	↓ (26–75%)	[11,12]
*I_to_*	↓ (50%)	↓ (40–64%)	[12,13]
*I_K1_*	↓ (45%)	↓ (27–57%)	[13,14]
*I_Kr_*	↓ (57%)	↓ (50–62%)	[14,15]
*I_Ks_*	↑ (~200%)	↑ (50–500%)	[13,14]
*I_NaCa_*	↓ (42%)	↓ (15–45%)	[16,17]
*I_NaK_*	↓ (25%)	↓ (25–50%)	[11]
*SERCA*	↑ (200%)	↑ (30–238%)	[20]

Note: Upward (↑) and downward (↓) arrows indicate increases and decreases, respectively, relative to the control condition. Values in parentheses denote the magnitude of the percentage change from the control value. For example, ↑200% represents a 200% increase (threefold the control value), whereas ↓50% represents a 50% reduction (0.5-fold the control value).

**Table 3:** Partial parameter settings for ventricular cell model [25].

Parameter	Description	Value
*R*	Gas constant	8.3143 J·K^−1^∙mol^−1^
*T*	Temperature	310 K
*F*	Faraday constant	96.4867 C∙mol^−1^
*C_m_*	Cell capacitance per unit surface area	2 μF/cm^2^
*S*	Surface-to-volume ratio	0.2 μm^−1^
*ρ*	Cellular resistivity	162 Ω·cm
*V_c_*	Cytoplasmic volume	16,404 μm^3^
*V_sr_*	Sarcoplasmic reticulum volume	1094 μm^3^
*K_o_*	Extracellular K^+^ concentration	5.4 mM
*Na_o_*	Extracellular Na^+^ concentration	140 mM
*Ca_o_*	Extracellular Ca^2+^ concentration	2 mM
*G_Na_*	Maximal *I_Na_* conductance	14.838 nS/pF
*G_k_*_1_	Maximal *I_K1_* conductance	5.405 nS/pF
*G_to_*, *epi, M*	Epicardial *I_to_* conductance	0.294 nS/pF
*G_to_*, *endo*	Maximal endocardial *I_to_* conductance	0.073 nS/pF
G_kr_	Maximal *I_Kr_* conductance	0.096 nS/pF
*G_Ks_ epi,endo*	Maximal EPI and ENDO cells cardial *I_Ks_* conductance	0.245 nS/pF
*G_Ks_*, *M*	Maximal M cell *I_Ks_* conductance	0.062 nS/pF
*p_KNa_*	Relative *I_Ks_* permeability to Na^+^	0.03
*G_CaL_*	Maximal *I_CaL_* conductance	3.98 × 10^−5^ mS/μF
*k_NaCa_*	Maximal *I_NaCa_*	1.000 pA/pF
*γ*	Voltage dependence parameter of *I_NaCa_*	0.35
*K_mCa_*	*Ca_i_* half-saturation constant for *I_NaCa_*	1.38 mM
*K_mNai_*	*Na_i_* half-saturation constant for *I_NaCa_*	87.5 mM
*k_sat_*	Saturation factor for *I_NaCa_*	0.1
*α*	Factor enhancing outward nature of *I_NaCa_*	2.5
*P_NaK_*	Maximal *I_NaK_*	1.362 pA/pF
*K_mK_*	*K_O_* half-saturation constant of *I_NaK_*	1 mM
*K_mNa_*	*Na_i_* half-saturation constant of *I_NaK_*	40 mM
*G_pK_*	Maximal *I_pK_* conductance	0.0146 nS/pF
*G_pCa_*	Maximal *I_pCa_* conductance	0.025 nS/pF
*K_pCa_*	Half-saturation constant of *I_pCa_*	0.0005 mM
*G_bNa_*	Maximal *I_bNa_* conductance	0.00029 nS/pF
*G_bCa_*	Maximal *I_bCa_* conductance	0.000592 nS/pF

**Table 4:** Quantitative cellular electrophysiological endpoints under control and I-CHF-motivated conditions.

Endpoint	Control	I-CHF-Motivated Scenario	Relative Difference
EPI APD_90_	280 ms	350 ms	+25%
ENDO APD_90_	300 ms	390 ms	+30%
EPI peak |*I_CaLa_*|	16.0 mA	8.8 mA	−45%
ENDO peak |*I_CaL_*|	14.0 mA	8.4 mA	−40%
Peak |*I_NaCa_*|	0.95 mA	1.95 mA	+105%

Note. Relative change was calculated as (I-CHF-control)/control × 100%. APD_90_, action-potential duration at 90% repolarization; EPI, epicardial cellular phenotype; ENDO, endocardial cellular phenotype.

**Table 5:** Quantitative metrics of the local model potentials shown in Figure 6.

Site	OW Peak-to-Peak (mV)	IW Peak-to-Peak (mV)	Max-|dφ/dt| Landmark (ms)	Difference vs. Site I (ms)
I	1.894	1.263	109.4	0.0
II	3.758	2.505	132.2	22.8
III	1.941	1.294	347.6	238.2

## Data Availability

No new data were generated or analyzed in this study. The mathematical models and computational methods are described in sufficient detail within the article to allow replication.

## Abbreviations

The following abbreviations are used in this manuscript:

AP	Action Potential
CHF	Chronic heart failure
ECG	Electrocardiogram
EPI	Epicardium
ENDO	Endocardium
FHN	Fitzhugh–Nagumo
PVCs	Premature ventricular contractions
I-CHF	Ischemic Chronic Heart Failure
OW	Outer-wall
IW	Inner-wall
